# Neuroprotective and Antioxidant Effect of Naringenin-Loaded Nanoparticles for Nose-to-Brain Delivery

**DOI:** 10.3390/brainsci9100275

**Published:** 2019-10-15

**Authors:** Shadab Md, Nabil A. Alhakamy, Hibah M. Aldawsari, Hani Zakaria Asfour

**Affiliations:** 1 Department of Pharmaceutics, Faculty of Pharmacy, King Abdulaziz University, Jeddah 21589, Saudi Arabia; nalhakamy@kau.edu.sa (N.A.A.); aldosarih@gmail.com (H.M.A.); 2 Department of Medical Microbiology and Parasitology, Faculty of Medicine, Princess Al-Jawhara Center of Excellence in Research of Hereditary Disorders, King Abdulaziz University, Jeddah 21589, Saudi Arabia; hasfour@hotmail.com

**Keywords:** antioxidant, chitosan, naringenin, nanoparticles, neuroprotection

## Abstract

Parkinson’s disease (PD) is a neurodegenerative disorder resulting in a decreased nigrostriatal availability of dopamine. Oxidative stress is one factor contributing to PD. Naringenin (NAR), a flavonoid, is a potent antioxidant shown to be beneficial in experimental PD. The clinical development of NAR has been hampered due to its low bioavailability resulting from gastrointestinal degradation, inefficient permeability, and low aqueous solubility. The objective of the present research was to formulate and characterize naringenin-loaded chitosan nanoparticles (NAR NPs) for nose-to-brain delivery. The cellular uptake, cytotoxicity, and neuroprotective effects of NAR NPs were determined using the SH-SY5Y cell line in vitro. NAR NPs were prepared using the ionic gelation method and characterized by zetasizer, transmission electron microscopy (TEM), and field emission microscopy (FESEM). The average particle size, polydispersity index (PDI), zeta potential, entrapment efficiency, and 24 h in vitro release profile were 87.6 ± 8.47 nm, 0.31 ± 0.04, 15.36 ± 2.05 mV, 91.12 ± 2.99%, and 54.80 ± 4.22%, respectively. The percentage NAR permeation through nasal mucosa from NPs was found to be 67.90 ± 0.72%. Cellular uptake of prepared NPs was confirmed by fluorescence microscopy. Neuroprotective activity of NAR NPs was evaluated through viability assays and by estimating reactive oxygen species (ROS) levels. NAR NPs showed enhanced neuroprotective ability and antioxidant effect against 6-OHDA-induced neurotoxicity in SH-SY5Y cells. However, animal studies are necessary to establish the potential of NAR NPs to be an effective carrier for the treatment of PD for nose-to-brain delivery.

## 1. Introduction

Parkinson’s disease (PD) is a neurodegenerative disorder that results in a decreased nigrostriatal availability of dopamine [1]. One of many factors contributing to the development of PD is oxidative stress. In the context of the pathogenesis of PD, oxidative stress is generated because of dysfunction of mitochondria and oxidative metabolism of dopamine [2]. Free radicals generated due to mitochondrial dysfunction could be responsible for the oxidative damage which further generates reactive oxygen species (ROS) resulting in a vicious cycle. The increase metabolism of dopamine, lipid peroxidation, and nitric oxide and reduced level of endogenous antioxidant enzymes such as glutathione (GSH) and superoxide dismutase in the brain could be responsible for neuronal death [3]. Although antioxidants have been shown to be highly effective in experimental PD [4,5,6], controlled clinical trials have shown no therapeutic effect [7]. However, there is an ongoing quest to identify antioxidant-based therapeutics for treatment of PD. Naringenin (NAR) is a flavonoid, potent antioxidant shown to be beneficial in experimental PD [4,8,9]. Three in vivo studies have demonstrated that naringenin is effective against experimental PD when administered systemically [4,5,6]. However, despite the therapeutic potential of naringenin shown in several animal models, the clinical development of naringenin has been hampered due to its low bioavailability as a result of gastrointestinal degradation, inefficient permeability, and low aqueous solubility [10,11]. As a result, various formulations have been investigated for naringenin to minimize drug degradation, increase bioavailability, achieve site-specific targeting, and to reduce side effects. These formulations include phospholipid complexes, cyclodextrin inclusion complexes, and polymeric nanoparticles [12,13]. However, these approaches have been only partially successful in increasing the solubility, permeability, and bioavailability. Moreover, none has resulted in site-specific targeting and high bioavailability of naringenin in the brain. The blood–brain barrier (BBB) also presents an impediment for the treatment of PD [14]. Thus, much effort has been made to develop ways to overcome the BBB to deliver antioxidants from blood to the brain. Nasal administration provides an alternative, non-invasive route for brain delivery circumventing the BBB. After intranasal application, drugs may be transported across the nasal membrane into the blood through the systemic circulation, from where they may cross the BBB by using specific transport mechanisms or due to their lipophilicity [15]. Drugs may also be reached directly into the cerebrospinal fluid (CSF) or the brain tissue due to the nature of the olfactory region of the nasal cavity [16]. The usual residence time for drugs in the nasal cavity is only 15–20 min because of mucociliary clearance, which restricts the amount of drug available for absorption [17]. The main strategy to improve drug delivery to the brain involves specific targeting of the olfactory region through bioadhesion to achieve drug release at the absorption site for prolonged periods of time [18]. An extension of this approach is to encapsulate drugs in biocompatible polymeric nanoparticles (NPs) for nasal administration. Polymeric NPs have been identified as drug carriers for intranasal route due to their strong interaction with mucous membranes and prevent drugs from enzymatic degradation [19]. Many researchers developed nanoparticulate drug delivery systems for nose-to-brain targeting all of which have their own advantages and disadvantages. Nanoparticles of bromocriptine, rivastigmine, venlafaxine, rotigotine have been prepared using different polymers; their biodistribution and drug targeting in the brain after intravenous or intranasal injection have been evaluated [16,17,18,19]. The novelty of our study is that no previous research has been published on the nose-to-brain delivery of naringenin for the treatment of PD using chitosan nanoparticles. Hence, the objective of the present research was to formulate and evaluate naringenin-loaded chitosan nanoparticles (NAR NPs). The cellular uptake, cytotoxicity, and neuroprotective effect of NAR NPs was determined in vitro using the SH-SY5Y cell line.

## 2. Material and Methods

Naringenin (NAR) powder (99% purity), chitosan (medium molecular weight, 75–85% deacetylated), pentasodium tripolyphosphate (TPP), MTT reagent, 6-OHDA, Hoechst 33342, coumarin-6, and Human neuroblastoma SH-SY5Y, American Type Culture Collection (ATCC) (ATCC.CRL-2266) were purchased from Sigma, USA. Dulbecco’s modified Eagle’s medium (DMEM, 12-604Q), fetal bovine serum (FBS), penicillin/streptomycin, trypsin-EDTA were purchased from Gibco, Gaithersburg, MD, USA. Reactive oxygen species (ROS) assay kit were purchased from Caymen Chemical (Ann Arbor, MI, USA).

### 2.1. Preparation of Blank and Naringenin-Loaded Chitosan Nanoparticles (NAR NPs) 

NAR NPs were produced using ionic gelation method as described in Tzeyung et al., (1). Chitosan was dissolved in aqueous acetic acid (1% v/v) solution and drug NAR in different concentration was dispersed in chitosan solution under continuous stirring to obtain final mixture of NAR with chitosan solution. Next, TPP solution as a crosslinker was added to the chitosan solution drop wise with constant stirring (600 rpm) at room temperature for 30 min. NPs were prepared after mixing of optimal concentration of polycation solution of chitosan and polyanion solution of TPP. The resulting NAR NPs formed were pelleted by centrifugation for about 40 min at 20,000 rpm. The prepared pellet was freeze-dried for further characterization and the supernatant solution was used to measure the entrapment efficiency. The drug free chitosan nanoparticles (blank NPs) were obtained using the same procedure.

### 2.2. Particle Size and Zeta Potential Analysis

The mean particle size and polydispersity index (PDI) or article size distribution of blank NPs and NAR NPs was determined using Zetasizer Nano-ZSP, (Malvern Instruments, Worcestershire, UK). The measurement was performed in triplicate. The Zeta potential of blank and NAR NPs was estimated with a Zetasizer Nano-ZSP. One milliliter of diluted blank NPs and NAR NPs were used for analysis. 

### 2.3. Transmission Electron Microscopy (TEM) and Field Emission Scanning Electron Microscopy (FESEM)

The actual size, shape, and morphology of NAR NPs were analyzed by TEM (Hitachi HT7700 TEM, Tokyo, Japan) and FESEM (Hitachi, Tokyo, Japan). For TEM analysis, NAR NPs were diluted and the diluted sample was placed on a formvar-coated copper grid and allowed to dry and the sample was visualized under the microscope. For FESEM analysis gold sputter technique was used and the shape and morphology of the NAR NPs were determined. 

### 2.4. In Vitro Release Study

In vitro drug release of NARNPs were determined by placing NPs in an activated dialysis bag in a beaker containing 50 mL of phosphate buffer solution (PBS, pH 7.4) under magnetic stirring at 100 rpm. The beaker was thermally controlled at 37 ± 0.5 °C. At predetermined time intervals, 2 mL of the samples were withdrawn and substituted with fresh buffer solution, and the absorbance was measured using UV spectrophotometry. 

### 2.5. Ex Vivo Permeation Study Using Goat Nasal Mucosa

Ex vivo nasal permeation studies of NARNPs and the NAR solution were carried out on goat nasal mucosa using Franz diffusion cell [1,17]. The drug solution and NAR NPs was placed in a donor compartment. The receptor compartment was filled with phosphate buffer saline (PBS) of pH 7.4 at 37 °C ± 0.5 °C. At each time point, 1 mL samples were withdrawn from the receptor chamber and an equal volume of PBS was added in chamber. The withdrawn samples were filtered and used for HPLC analysis. 

### 2.6. Cytotoxicity Studies

The cell viability was measured by calorimetric MTT [3-(4,5-dimethylthazol-2-yl)-2-5-diphenyl tetrazolium bromide] assay in vitro. SH-SY5Y cells seeded at 3 × 104 cells/well (100 μL) in 96-well plates and incubated at 37 °C or 24 h. After culturing for 24 h, the cells were treated with free NAR solution and NARNPs at different concentrations ranging from 5–100 µg/mL. NAR was dissolved in dimethylsulfoxide (DMSO) and then diluted in culture medium to obtain a final concentration of DMSO (0.1%). Untreated cells were used as control and the plates were incubated in a humidified incubator having 5% CO2 and 95% O2 at 37 °C for another 24 and 48 h. The MTT assays were performed by adding 20 μL of MTT solution (5 mg/mL in PBS) into each well, followed by 4 h incubation. After that all medium was carefully aspirated from the wells, 200 μL of DMSO was added to each well, and the plates were shaken for 15 min while being protected from light. The absorbance of each well was measured at 570 nm using a micro-plate reader (TECAN, Switzerland), using the wavelength of 630 nm as reference. Cell viability was expressed as percentage viable cells.

Cell viability = Absorbance of experimental well/Absorbance of negative control well × 100

### 2.7. Cellular Uptake Studies

Qualitative cellular uptake of NAR NPs was determined using fluorescence microscopy using coumarin-6, which was encapsulated into NPs instead of NAR as a fluorescent probe to track the NPs [20]. Coumarin-6 solution was prepared by dissolving coumarin-6 powder in absolute ethanol. Seeded into 6-well plate and incubated for 24 h were 3 × 105 cells. On day 2, the medium was carefully aspirated. Then, the cells were treated with 0.5 μg/mL of coumarin-6 solution and coumarin-6 labeled NPs and incubated for 4 h. After incubation, the cells were washed three times with cold PBS and fixed with 4% paraformaldehyde for 10 min at room temperature. The cells were further incubated with Hoechst 33342 for 10 min in order to stain the cell nucleus. Then, the cells were washed three times with cold PBS and visualized using fluorescence microscopy.

### 2.8. Neuroprotective and Cellular Reactive Oxygen Species (ROS) Activity of NAR NPs

The SH-SY5Y cells were seeded at 3 × 10^4^ cells/well in 96 well-plate and incubated for 24 h. The medium was carefully aspirated, and the cells were treated with NAR solution, NAR NPs, and blank NPs at a concentration of 10 µg/mL for 4 h. For negative and positive control wells, complete culture medium was added. After 4 h incubation, the medium containing formulations was aspirated and 100 μL of 45 µM 6-OHDA was added in each well and incubated for 24 h. After 24 h incubation, all medium was carefully aspirated from the wells, 200 μL of DMSO was added to each well, and the plates were shaken for 15 min while being protected from light. The absorbance of each well was measured at 570 nm using a micro-plate reader (TECAN, Mannedorf, Switzerland), using the wavelength of 630 nm as reference. Cell viability was expressed as percentage viable cells. The effect of NAR NPs on 6-OHDA-induced production of ROS was assessed using the DCFDA-cellular reactive oxygen species detection assay kit as per the manufacturer’s instruction. Negative controls consisted of cells treated with 0.1% DMSO (vehicle control, VC) and cells treated with 6-OHDA without prior treatment with NAR solution and NAR NPs are positive control.

### 2.9. Statistical Analysis

Statistical analysis was carried out by Student’s *t* test and analysis of variance (ANOVA) followed by post hoc Tukey’s tests using PASWs 18 (SPSS software, Chicago, IL, USA). All results were expressed as mean ± SD. The *p* values (*p* < 0.05) values were considered statistically significant.

## 3. Result and Discussions

### 3.1. Development and Optimization of NAR NPs

**Figure 1:**
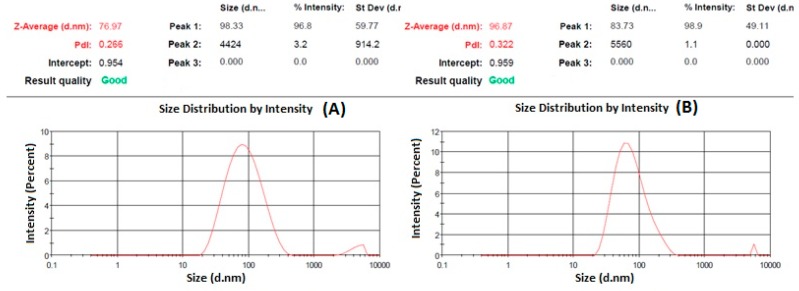
Represent particle size and particle size distribution of blank nanoparticles (NPs) (**A**) and naringenin-loaded chitosan nanoparticles (NAR NPs) (**B**) measured by zetasizer.

In order to develop NAR NPs, an ionic gelation method was used [1]. For this purpose, CS was used at various concentration ranges (0.5–2 mg/mL). The tripolyphosphate was used at a concentration of 0.5 mg/mL. From various hit and trial methods, stirring speed (600 rpm) and stirring time (30 min) were fixed for preparation of NPs. Different polymer concentration, TPP volume, and concentration of drug were varied to optimize best formulation. As per previous literature report for nose-to-brain delivery, optimum particles size should be less than 200 nm. Particle size plays an imperative role in cell uptake, drug release, nasal permeation, and biodistribution studies [16]. Therefore, formulation process variables were optimized to obtain NPs with desirable size range. All these process variables showed major effect on particle size, PDI, zeta potential (ZP), and entrapment efficiency as shown in Figure 1 and Table 1. Blank NPs were prepared after various preliminary trials and varying optimization parameters (data not shown). The optimized blank NPs at 0.5 mg/mL of chitosan, TPP volume (4 mL), stirring speed 600 rpm, and stirring time 30 min had an average particle size of 60.80 ± 2.74 nm, a PDI of 0.225 ± 0.03, and a ZP of 20.6 ± 0.25. It was also demonstrated that at low TPP volume (1–2 mL), larger NPs were observed due to the reduced quantity of TPP available to cross link with chitosan molecules. Further increases in volume from 3 to 5 mL resulted in a decrease in particle size and PDI (data not shown) due to increased crosslinking between chitosan and TPP. However, further increases in volume from 6–7 mL resulted in a sudden increase in particle size and the formation of aggregates, due to saturation of the chitosan chain with TPP and excess TPP precipitated in larger aggregates [17,21]. These optimized blank NPs were further used for the preparation of NAR NPs. NAR NPs were prepared and the effect of increases in chitosan concentration on average particle size, PDI, and ZP were observed as shown in Table 1. The average particle size, PDI, and ZP were increased gradually from 73.9 to 567.8 nm, 0.25 to 0.79, and 18.9 to 32.8 mV, respectively, with increases in chitosan concentration from 0.5–2 mg/mL as shown in Table 1. This was in agreement with the data reported previously Md et al. [17]. This may be explained by more chitosan chains per volume at higher chitosan concentrations; this would lead to insufficient electrostatic repulsion, stronger intermolecular hydrogen bonding attraction, and decreased intermolecular distance resulting in the formation of larger particles with non-uniform particle size distribution [21,22]. Formulation code F1 (PS: 93.9 ± 12.9; PDI: 0.25 ± 0.08; and ZP: 18.9 ± 2.5) was selected among all other formulations shown in Table 1 because of small particle, narrow particle size distribution, and acceptable zeta potential. Formulation F1 was further utilized to optimize the drug concentration. The effect of different drug concentrations on average particle size, PDI, ZP, and the entrapment efficiency percentage (%EE) were observed (Table 2). When the drug concentration was increased from 0.1 to 1 mg/mL, the average particle size of NAR NPs increased from 72.7 to 857.7 nm, with an increase in PDI. This was in agreement with a previous report [1], the increase in NP size could be due to reduced CS/TPP interaction. On the other hand, initial increases in NAR concentration (0.1 to 0.25 mg/mL) increased the EE (entrapment efficiency) from 84.45 ± 5.25 to 91.12 ± 2.99%, although this did not reach statistical significance (*p* > 0.05). Further increases in drug concentration (0.5 to 1 mg/mL) led to a significant decrease (*p* ≤ 0.05) in %EE from 74.6 ± 4.09 to 59.19 ± 5.36% (Table 2). The reason for this decrease could be due to more NAR molecules being electrostatically adsorbed onto the surface of the chitosan, which was easily separated from NPs due to centrifugation [17]. Similar findings were reported by Wu and coworkers [23] who showed that the %EE of ammonium glycyrrhizinate in CS NPs decreased with increasing drug loading concentration. The criteria for selecting an optimized formulation of NAR NPs were lower particle size, narrow PDI, and higher ZP and %EE. In our study, formulation code F1N1 and F1N2 did not show any significant changes in formulation characteristics; therefore, any formulation could be selected. Hence, in this study we have selected F1N2 code for further in vitro study.

### 3.2. Characterization of NAR NPs

**Figure 2:**
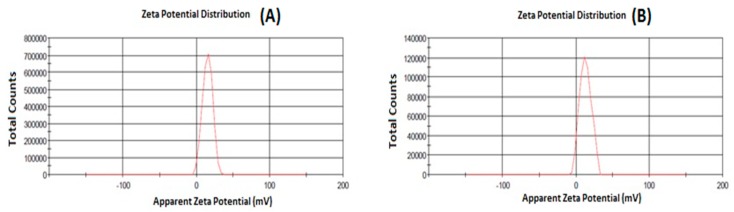
Represent zeta potential of blank NPs (**A**) and NAR NPs (**B**) measured by zetasizer.

**Figure 3:**
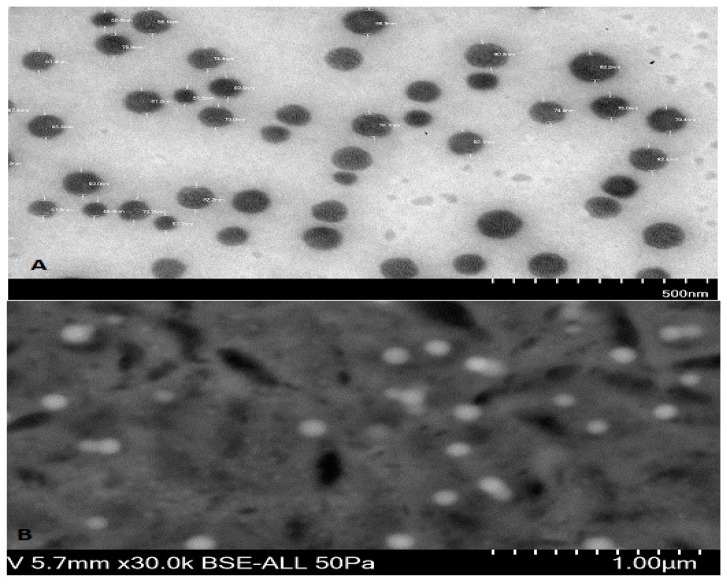
Microphotograph of NAR NPs by (**A**) Transmission Electron Microscope (TEM) and (**B**) Field Emission Scanning Electron Microscope (FESEM).

Particle size is one important parameter requiring to be optimization for nose-to-brain delivery, because smaller particles increase the rate of drug absorption, and more drug can be transported through the olfactory region in the brain [21]. The particle size, PDI, and zeta potential were characterized by zetasizer as shown in Figure 1 and Figure 2. The hydrodynamic particle sizes of blank NPs and NAR NPs were 76.97 nm and 96.87 nm, respectively. The differences between the size of the blank NPs and NAR NPs could be due to the presence of the drug (Figure 1A,B). The respective particle size distribution of blank NPs and NAR NPs were 0.266 and 0.322, exhibiting narrow particle size and unimodal distribution (Figure 1A,B). The zeta potential represents an index for particle stability and the positive zeta potential (15 mV) indicated stability of the prepared NAR NPs (Figure 2A,B). The positive value for zeta potential could be due to the cationic nature of chitosan and the presence of residual amino groups which are not neutralized by interaction with TPP molecules [24]. The positive charge of NAR NPs facilitates mucoadhesion and a prolonged residence time, as well as reduced mucociliary clearance of NPs from the nasal cavity [24]. The actual particle size of the prepared NAR NPs formulations was studied using TEM analysis. The images (Figure 3A) showed that the prepared formulations were in the nanosize range. The particles were spherical, well separated, uniformly distributed, and most of them were smaller than 60 nm (Figure 3A). The zetasizer studies showed hydrodynamic particle sizes considerably larger than the particle size determined using TEM. This difference could be due to the dry state of the TEM measurements [19]. TEM images showed no aggregation of particles. The FESEM images shows that the NAR NPs were spherical, and had a smooth surface and morphology. There was little aggregation of the particles due to freeze drying (Figure 3B).

### 3.3. In Vitro Drug Release

**Figure 4:**
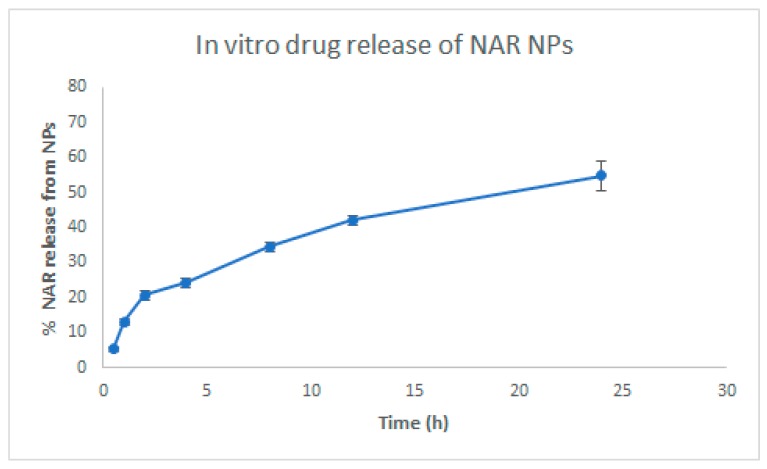
In vitro release studies of NAR NPs in release medium at pH 7.4 over 24 h. Values are expressed as mean ± SD (*n* = 3), three independent experiments performed in triplicate.

The in vitro release of NAR from the NPs is shown in Figure 4. NAR release from NPs in vitro showed an initial rapid release (20.64 ± 1.35%) for 2 h, followed by a slow release (54.80 ± 4.22%) for 24 h. The initial rapid release could be due to the presence of drug molecules on the surface of the NPs [25]. The slow and sustained release of NAR from NPs was due to the presence of entrapped drug in the matrix. The slow drug release from the matrix could be due to penetration of fluid media into the matrix by diffusion mechanism, thus converting the polymer matrix system into a rubbery state which is then degraded or eroded [25]. All these mechanisms take place simultaneously leading to controlled release of NAR from NPs.

### 3.4. Nasal Permeation Study

**Figure 5:**
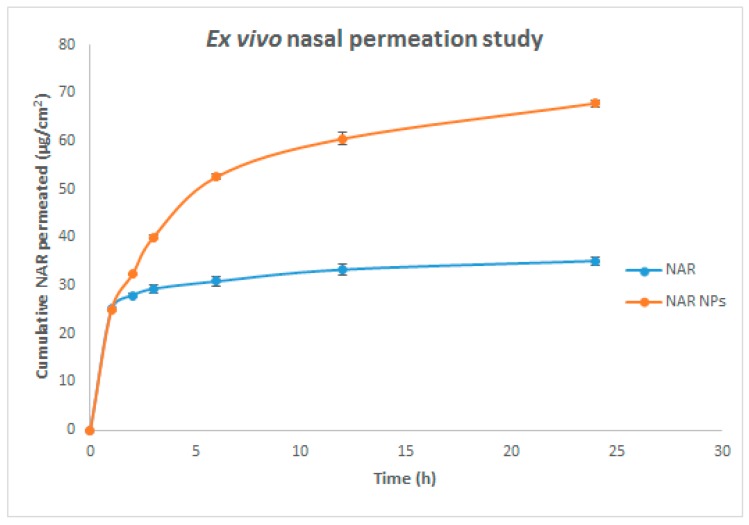
Ex vivo nasal permeation of NAR NPs and NAR solution in in PBS at pH 7.4. Values are expressed as mean ± SD (*n* = 3), three independent experiments performed in triplicate.

Nasal permeation studies were performed using goat nasal mucosa to determine comparative nasal permeation of NAR from NAR solution alone and from NAR NPs. The NAR permeated through the nasal mucosa from NAR NPs was 67.90 ± 0.72%, whereas only 35.14 ± 0.92% was permeated from NAR solution (Figure 5). The steady-state flux of NAR solution and NAR NPs through the nasal mucosa was 0.015 ± 0.001 µg cm^−2^h^−1^ and 0.071 ± 0.002 µg cm^−2^h^−1^, respectively; the enhancement ratio for flux was 4.72 ± 0.14. The reduced permeation of NAR solution could be due to the crystalline nature of NAR and the lipophilic nature of the nasal mucosa acting as a rate-limiting membrane through which NAR did not diffuse well as reported in previous literature [1,17]. The high permeation of NAR from NAR NPs could be due to the presence of residual amino groups in the chitosan resulting in the formation of positive NPs which interact with sialic acid residues in the cell membrane. These electrostatic interactions induce a transient opening in the tight junctions of nasal mucosa, which enhances the paracellular transport and permeation across the nasal surface [1,17].

### 3.5. Cellular Uptake Studies

**Figure 6:**
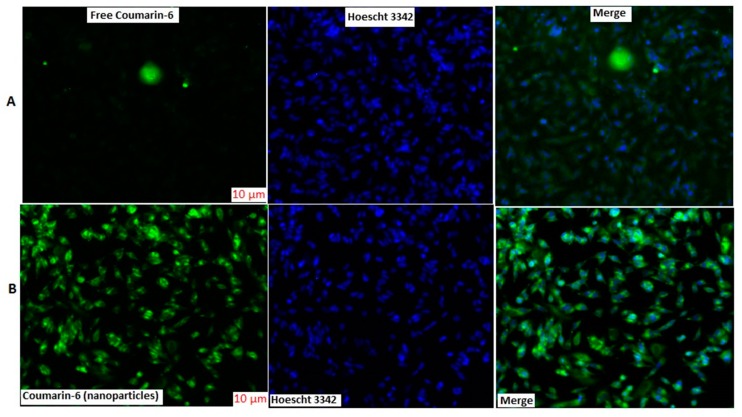
Fluorescence microscopy images of cellular uptake of coumarin-6 NPs and coumarin-6 solution in SH-SY5Y cells. The scale bar for (**A**,**B)** is 10 μm.

Cellular uptake was qualitatively measured using fluorescent microscopy. The fluorescence intensity of coumarin-6 solution was lower than coumarin-6 loaded NPs (Figure 6). The cell nuclei were stained with Hoechst 3342 to visualize the distribution of nanoparticles. The NPs were well distributed in cytoplasm surrounding the nuclei and did not penetrate the nucleus as observed by green fluorescence intensity (Figure 6). The uptake pattern of NPs showed better penetration, internalization, sustained retention, and better absorption inside the cell, indicating an efficient delivery system [20,26,27,28].

### 3.6. Cytotoxicity and Neuroprotective Studies Against 6-OHDA Induced Neurotoxicity

**Figure 7:**
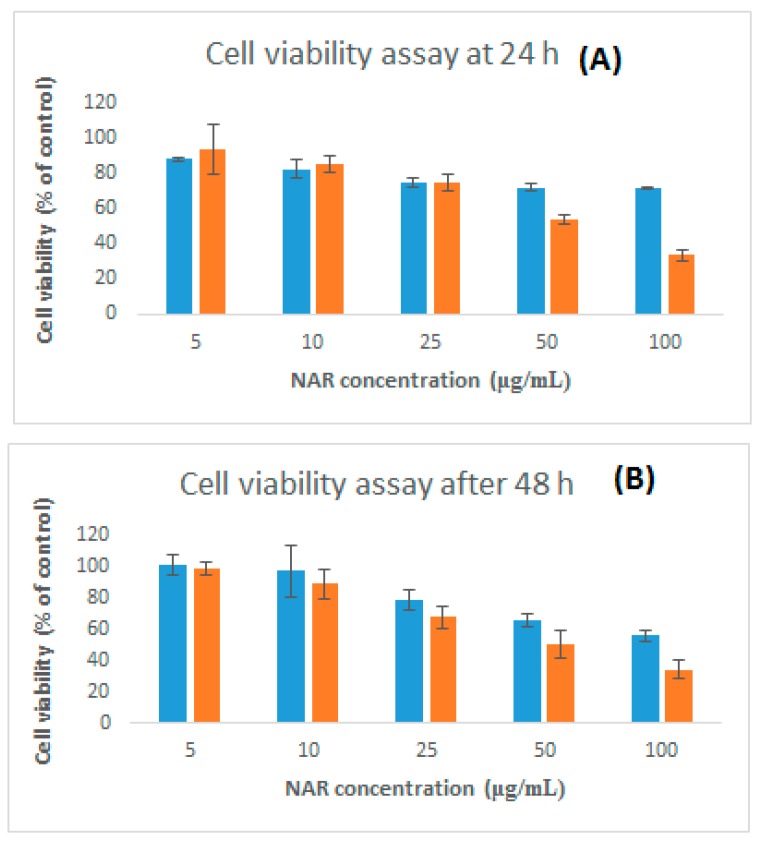
Dose and time dependent in vitro cytotoxicity of free NAR and NAR NPs on SH-SY5Y cells using MTT assay. Cell viability assay at 24 h (**A**) and 48 h (**B**). Control consisted of cells treated with 0.1% DMSO (vehicle control, 100 % cell viable). Values are expressed as mean ± SD (*n* = 3), three independent experiments performed in triplicate.

The cytotoxicity of NAR and NAR NPs at different concentrations (5–100 μg/mL) and times (24 h and 48 h) was investigated using the SH-SY5Y cell line (Figure 7). Free NAR showed no significant toxicity at all the concentrations tested for 24 and 48 h as compared to control group (100 ± 8.2%; *p* > 0.05). NAR NPs showed no toxicity in the concentration range 5–25 μg/mL at 24 and 48 h; however, NAR NPs exhibited significant cytotoxicity at high concentrations (50–100 μg/mL at 24 and 48h, *p* < 0.05). Treatment with 100 μg/mL of NAR NPs resulted in only 33.83% cell viability at 24 h. The cytotoxicity of NAR NPs at higher concentrations is in agreement with previous studies [28]. These results suggest that the NPs were nontoxic below a concentration of 50 μg/mL; this suggested their safety in normal cells and that lower concentrations could be used safely in further studies. We next investigated if a low concentration of NAR NPs could protect the SH-SY5Y cells against 6-OHDA-induced neurotoxicity (Figure 7A). Exposure to 6-OHDA resulted in a significant decrease in the viability of neurons treatment (49.18 ± 3.19%) as compared to the negative control group (100 ± 12.59%) (*p* < 0.01). Pretreatment with NAR solution, the cell viability was 54.17 ± 1.60% which was not significantly different (*p* > 0.05) from the positive control (6-OHDA alone). However, pretreatment with NAR NPs significantly increased cell viability on (73.36 ± 4.27%) as compared with 6-OHDA alone (positive control) (*p* < 0.01) and NAR solution (*p* < 0.05). Blank NPs (50.73 ± 3.56%) did not shown any significant effect (*p* > 0.05) against 6-OHDA-induced neurotoxicity, so they were not included for further studies [19,20]. This indicates that despite the weakly cytotoxic nature of NAR NPs, they can effectively protect the cells against 6-OHDA induced neurotoxicity [28,29,30].

### 3.7. ROS Levels in SH-SY5Y Cells on Pretreatment with NAR NPs

**Figure 8:**
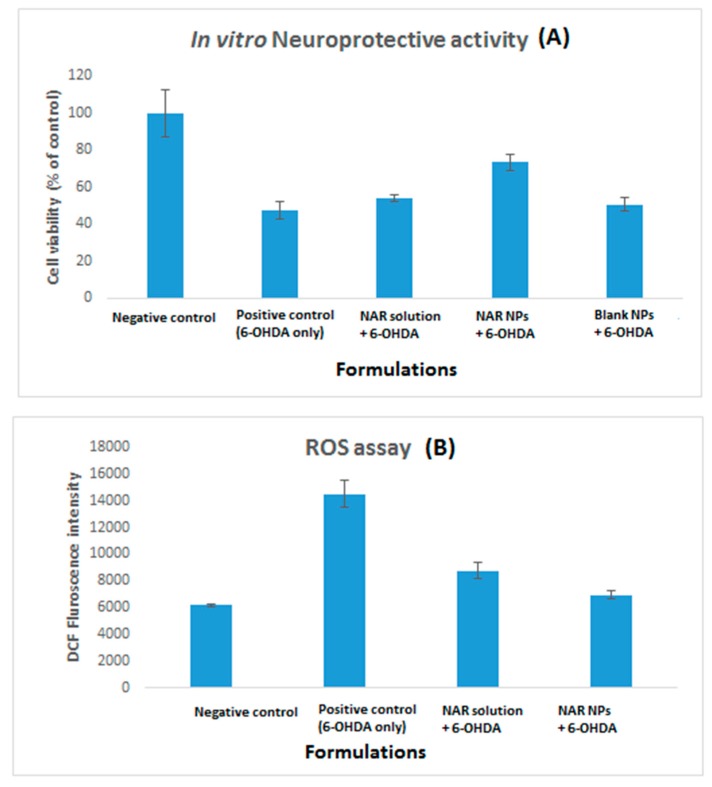
(**A**) Neuroprotective activity and (**B**) reactive oxygen species (ROS) levels of NAR solution, NAR NPs, and placebo NPs were determined in SH-SY5Y cells using MTT assay on 6-OHDA induced neurotoxicity. Negative controls consisted of cells treated with 0.1% dimethylsulfoxide (DMSO) (vehicle control, VC) and cells treated with 6-OHDA alone without prior treatment with NAR solution and NAR NPs are positive control. Data was represented as % cell viability in a bar graph. Viability of control cells was 100%. Data are expressed as mean ± SD (*n* = 3). The data were analyzed using one-way ANOVA followed by Tukey’s post hoc test. Percentage of viable cells in each group was compared with VC, *p* < 0.05 and *p*< 0.01 indicates a significant and highly significant difference respectively.

6-OHDA induces neurotoxicity via enhancing ROS levels. Antioxidant activity of NAR NPs was determined through pretreatment of 6-OHDA treated cells with NAR solution and equivalent NAR NPs individually, followed by a 24 h treatment with 45 µM 6-OHDA. ROS levels were significantly (*p* < 0.01) higher in cells induced with positive control (14,529 ± 1004 unit) as compared to negative control (6181 ± 81 unit) (Figure 8B). The samples pretreated with NAR solution (8753 ± 598 unit) and NAR NPs (6944 ± 315 unit) demonstrated significantly lower (*p* < 0.01) ROS level compared to the positive control group. However, NAR NPs produced a significantly greater reduction in ROS level than NAR solution. These results confirmed the antioxidant activity of NAR NPs in SH-SY5Y cells [28,29,30]. As we know from the previous literature [29,30], dopamine agonists induce antioxidant activities via increasing the activities of radical-scavenging enzymes or by direct scavenging of free radicals. These results show an improvement in antioxidant activity of NAR NPs in SH-SY5Y cells as compared to NAR itself; this is probably due to the small size of the NAR NPs and their better cellular uptake [29,30].

## 4. Conclusions

In summary, NAR NPs were successfully developed, optimized, characterized, and evaluated. The NAR NPs were optimized on the basis of various process variables such as CS concentration, TPP volume, and NAR concentration. NAR NPs were evaluated using Zetasizer, TEM, FESEM, and the desired characteristics of small particle size, low PDI, positive zeta potential, and high entrapment efficiency were achieved for nose-to-brain delivery. The positive zeta potential indicated stability of NAR NPs due to the presence of CS. The in vitro drug release profile showed controlled release of NAR from the NPs. The amount of NAR permeated and steady-state flux through nasal mucosa was higher for NAR NPs as compared to NAR solution. NAR NPs showed no cytotoxicity at lower concentration (≤50 μg/mL). NAR NPs showed enhanced neuroprotective ability and antioxidant effect against 6-OHDA-induced neurotoxicity in SH-SY5Y cells. The improvement of cellular uptake of NPs by SH-SY5Y cells was demonstrated by fluorescence microscopy analysis. Taken together, these results show that NAR NPs can be used as an effective carrier for the treatment of PD by the intranasal route. However, pre-clinical and clinical studies are necessary to perform in future to establish the potential of NAR NPs in the market as a new way for the treatment of PD.

## Figures and Tables

**Table 1:** Effect of increase in chitosan concentration on particle size, polydispersity index, and zeta potential.

Formulation Code	Concentration of Chitosan (mg/mL)	PS ± SD (nm)	PDI ± SD	ZP ± SD (mV)
F1	0.5	93.9 ± 12.9	0.25 ± 0.08	18.9 ± 2.5
F2	1	127.2 ± 25.7	0.34 ± 0.08	23.6 ± 5.0
F3	1.5	302.8 ± 15.3	0.73 ± 0.18	28.6 ± 3.4
F4	2.0	567.6 ± 80.5	0.79 ± 0.17	32.8 ± 3.3

Formulation code F1 to F4 consist of fixed drug concentration (0.25 mg/mL); TPP volume 4 mL, stirring speed 600 rpm; stirring time 30 min. NAR: Naringenin; PDI: polydispersity index; PS: particle size; ZP: zeta potential; SD: Standard deviation. Values are expressed as mean ± SD (*n* = 3), three independent experiments performed in triplicate.

**Table 2:** Effect of increase in naringenin concentration on particle size, polydispersity index, zeta potential, and entrapment efficiency.

Formulation Code	Concentration of NAR (mg/mL)	PS ± SD (nm)	PDI ± SD	ZP ± SD (mV)	%EE ± SD
F1N1	0.1	72.7 ± 4.95	0.29 ± 0.02	19.66 ± 2.53	84.85 ± 5.25
F1N2	0.25	87.6 ± 8.47	0.31 ± 0.04	15.36 ± 2.05	91.12 ± 2.99
F1N3	0.50	385.1 ± 16.4	0.59 ± 0.05	12.53 ± 0.73	74.6 ± 4.09
F1N4	1.0	857.7 ± 23.6	0.68 ± 0.06	18.8 ± 0.70	59.1 ± 5.32

Formulation code F1N1 to F1N4 consist of fixed chitosan concentration (0.5 mg/mL); TPP volume 4 mL, stirring speed 600 rpm; stirring time 30 min. EE: entrapment efficiency; NAR: Naringenin. Values are expressed as mean ± SD (*n* = 3), three independent experiments performed in triplicate.
